# Correlation Analysis Model of Social Capital and Innovation Performance Based on Knowledge Mapping

**DOI:** 10.1155/2022/2138200

**Published:** 2022-06-06

**Authors:** Yuanyi Ding

**Affiliations:** Nanjing Audit University, Nanjing, Jiangsu 211815, China

## Abstract

In this paper, the correlation between social capital and innovation performance is analyzed using a knowledge graph approach, and a correlation analysis model is designed for practical use. Given the advantages of knowledge graph technology and the fact that most recommendation models fail to make full use of the correlation between knowledge graph and recommended items, this paper proposes a cross-attention fusion-based knowledge graph recommendation algorithm (CAKR). The CAKR model contains a cross-fusion module, a recommendation task module, and a knowledge graph embedding task module, and the inputs of the two task modules are alternately learned in a low-dimensional space by the cross-fusion module. The input of the two task modules is alternately learned in the low-dimensional space by the cross-fusion module to interact with the two embedding vectors of items and entities, and then, the obtained feature vectors are fed into the relevant task modules, respectively, and then, the results are calculated by the respective prediction functions. There is a mediating role of knowledge management capabilities in the innovation ecology perspective between social capital and innovation performance of SMEs. Among them, it fully mediates the relationship dimension of technical social capital, the cognitive dimension of institutional social capital, the relationship and cognitive dimension of market-based social capital, and innovation performance, and partially mediates the structural and cognitive dimension of technical social capital, the structural and relationship dimension of institutional social capital, and innovation performance. The relationship between knowledge management capability and innovation performance is positively mediated by environmental dynamics in the innovation ecology perspective, and the positive effect of knowledge management capability on innovation performance is more significant when the environmental dynamics are stronger, and vice versa. Finally, based on the results of the empirical study, some management suggestions are provided on how SMEs can reasonably utilize social capital to enhance their innovation performance.

## 1. Introduction

At present, due to the complex and diverse consumer demands, the drastic dynamic changes in the market environment, and the constant compression of the current business competition space, the cooperation mode among various innovation agents has changed from simple bilateral cooperation or vertical integration to a complex multilateral dynamic network cooperation mode. In the evolution process of long-term cooperation and interdependence of these innovation subjects, an innovation ecosystem like the mutual influence and interdependence of multiple communities in nature is formed, which realizes the leap from the engineering mechanized innovation system to the ecological organic innovation ecosystem [1]. The continuous development of the real economy requires the effective allocation of resources, which in turn leads to the formation of the innovation ecosystem. Based on the common innovation goal, each innovation subject enjoys a common environment, rights, resources, and advantages in the innovation ecosystem. Based on achieving individual forward development, they maintain the orderly operation of the overall system as a part of the innovation ecosystem, thus creating a common value that cannot be realized by individual organizations. Since innovation is characterized by uncertainty, complexity, and risk, enterprises cannot rely on their strength alone, but also need to introduce complementary and heterogeneous resources through social relationships with the external environment, and promote information exchange and technology sharing among enterprises. Many enterprises have begun to use alliances and social relationships to seek cooperation to obtain useful resources and valuable information to compensate for their shortcomings in innovation to reduce losses caused by the negative characteristics of enterprise innovation. The board of directors, as the most important management team of a company, is the bridge between the company and the outside world [2]. The social capital of the board of directors can obtain resources for the enterprise, can access to high-quality information technology, and is the key for the enterprise to establish relations with the outside world. The richer the social capital of the board of directors, the better it is for the enterprise to obtain reliable information resources and promote mutual learning among enterprises, thus increasing the richness of the enterprise's knowledge, enriching the enterprise's innovation elements, and thus improving the enterprise's innovation performance [3].

The development of social networks makes the connection between social subjects closer, which enables enterprises to have more external innovation resources and access to innovative ideas and talents. Social capital refers to the association and trust between social agents, which is expressed in the form of a relationship network and is the resource brought by the position of social agents in the social network [4]. By using social capital, enterprises can strengthen their interpersonal relationships with other network members and thus gain access to innovation resources and innovation information [5]. This facilitates enterprises to make timely and effective response strategies to changes in the external innovation environment. In addition, collaborative research and development with external research institutions can enhance the enthusiasm of enterprises to carry out innovation activities and improve the output rate of innovation inputs [6]. Therefore, the social capital accumulated by enterprises can strengthen the communication and cooperation with external organizations, enhance the enterprises' resources and information acquisition ability, and thus promote their innovation performance. The enterprise is the main body of innovation resource allocation. Enterprises are the allocators, ultimate realizers, and beneficiaries of innovation resources. According to their own innovation value chain, enterprises rationally choose their innovation strategies, allocate innovation resources, and accept the test of the market law. Enterprises can choose to achieve market success through marketing innovation from the lower end of the innovation chain, or they can choose to digest and absorb the technology of other enterprises and innovate by developing improved products from the middle and lower end. Firms have different capitals, capabilities, and strategies for doing business, and their innovation autonomy should be respected. With the massive inflow of innovation resources, the allocation of innovation resources can only be done effectively by relying on the many enterprises in a competitive market. Government departments should not and can hardly replace enterprises in effectively completing complex innovation decisions.

Social capital ties enterprises and external organizations together, can promote technological innovation activities, and provides certain material support for the improvement of their technological innovation performance. Therefore, this paper compares the previous literature, studies the influence path of social capital on the technological innovation performance of enterprises, analyzes how social capital affects the technological innovation performance of enterprises, helps enterprises to build and improve social networks in a targeted manner, guides enterprises to accumulate and cultivate social capital, and has certain guiding significance for improving the technological innovation performance of enterprises. With the progress of science and technology and the shortening of the technology renewal cycle, technological innovation has become an important part of market competition. Technological innovation is a process of knowledge creation, which is dynamic, complex, and networked, and social capital exists in social networks, which can promote technological innovation activities and improve technological innovation performance. Although companies have understood that social capital can contribute to their technological innovation performance, they are unable to target the role of social capital due to the lack of research on the transmission mechanism between the two. In this paper, we select a single company to systematically illustrate how social capital affects technological innovation performance, which can guide companies to proactively cultivate social capital and bring out its contribution to technological innovation performance.

## 2. Related Works

The formulation of the concept of social capital is representative and has been followed by some subsequent scholars. He considers social capital as the ability of subjects in a social network to obtain resources through network relationships. Meanwhile, the social capital of an organization includes the social capital of the organization itself and the members within the organization [7]. By summarizing the studies of foreign scholars on the definition of social capital, it is found that social capital contains elements such as trust, norms, cooperation, and network relationships, and it is proposed that social capital is a resource that exists in the social network formed by organizations based on trust and can bring economic benefits to enterprises [8]. After reviewing the previous studies on social capital, he found that it is difficult to unify the definition of social capital because each scholar defines social capital according to their own research needs. He defines social capital from a systemic perspective and argues that social capital is widely present in the relevant roles of social structures and that it can significantly improve the welfare of social agents [9]. He proposes the idea of applying the concept of social capital in enterprises, which refers to the collection of explicit and potential resources embedded in the network structure of the enterprise, and the manipulation and effective use of these resources to achieve its goals and target activities for the enterprise [10]. As most enterprises either lack original innovation, or are at the middle or low end of the industrial value chain, or are restricted in key technologies and basic components, the low level, high cost, and low income of innovation activities coexist, and enterprises have to face the problems of low value-added products, low market recognition, high sunk costs and high conversion costs, and the “late-development disadvantages” far outweigh the “late-development advantages.” It can be conceptualized separately in terms of internal and external social capital of the enterprise: External social capital of the enterprise is the ability to obtain scarce resources from outside. Social network capital is the intermediary in the expected coming shadow of the development of the enterprise, through the integration of financial and human capital, reaching consensus on rules and cooperation through historical transactions resounding the development process of the enterprise [11].

Under the development of modern technology, the contact nodes of social capital are more connected through information, and the information transfer through the network can make information more intensive and communication freer. Internal social capital refers to the ability of enterprises to optimize internal resources and improve production efficiency [12]. Specifically, enterprises connect employees and related departments at the level of mutual trust and norms to improve the efficiency of cooperation among various physical elements and employees of the enterprise and increase the return on efficiency. DBpedia is used to automate the extraction of structured information from Wikipedia to build large-scale knowledge graphs by defining knowledge extraction templates by members of the Internet community [13]. As the main source of DBpedia knowledge, the representation and writing of knowledge in Wikipedia are not always consistent, because people understand and express knowledge differently. To solve the problem of inconsistent representation brought by extraction, DBpedia is built using a mapping mechanism to achieve uniformity and consistency in knowledge description. The knowledge in Wikipedia is constantly updated and iterated, and to keep pace with it, DBpedia developed the DBpedia Live system to regularly monitor the content updates of Wikipedia [14]. Relationships follow the principle of balanced transactions, which means that the interaction between people is mutual. Each person has a different weighting in your life, there are long lines and short lines in relationships, some people will always become very important to you, and then, these are the people you need to maintain with the utmost care for the rest of your life. Let others treat you how you want to be treated, and please treat them in the same or even better way. The value of relationships lies in the scarcity of each person's time. Investing in time requires us to be clear and purposeful, and we need to invest more in those who deserve it, while reducing unnecessary social waste.

Researchers are constrained by objective conditions and research objectives to select samples from different countries, regions, or industries, and these differences in scenarios may lead to changes in the impact of social capital on manufacturing technology innovation performance [15]. Due to the different research designs, researchers will choose different technological innovation performance measurement methods and technological innovation performance measurement dimensions, and these methodological differences may also lead to the emergence of different results on the impact of social capital on manufacturing technological innovation performance. This study hopes to integrate different dimensions of social capital using meta-analytic techniques in the hope of gaining a clear understanding of the relationship between social capital and manufacturing technology innovation performance and to explore which situational and methodological factors moderate the relationship between the two to provide a reasonable explanation for the controversial nature of the current study and to enhance the application of social capital in realistic technology innovation management activities in manufacturing.

## 3. Knowledge Mapping of Social Capital and Innovation Performance Construction

As a hot topic of research in recent years, knowledge mapping has been widely used in various fields of computer disciplines such as recommendation systems, information retrieval, data mining, and visual analytics. The artificial intelligence technology that is closely linked with human knowledge is natural language processing, which can further understand the specific meaning of people's language with the background knowledge provided by knowledge graphs. Creative ability consists of a variety of competencies, which include the ability to learn, analyze, synthesize, imagine, critique, create, solve problems, practice, organize and coordinate, and integrate multiple competencies. Creative abilities have four main characteristics. Practically, the difference between innovation and invention lies in its application and the realization of the value of the results of creative inventions. With perseverance, innovation is a complex process involving the innovator's own abilities and the social environment. To succeed requires repeated trials and explorations, and success is only possible through perseverance.

**Figure 1:**
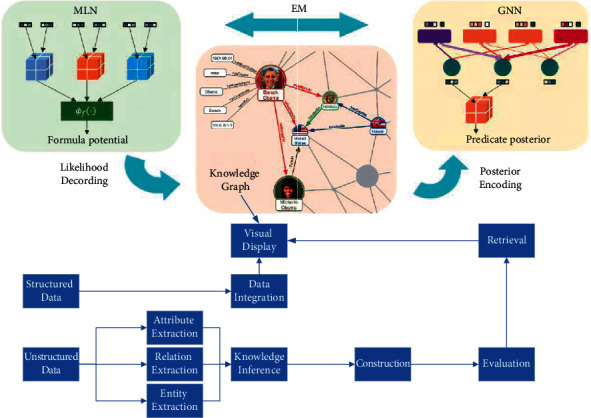
Knowledge graph construction process.

The same is true in the recommendation system scenario, where the background knowledge is used to understand the labels, attributes, and relationships of items with other items to make better choices [16]. The process of building a knowledge graph is shown in Figure 1. Because of the great benefits of knowledge graphs, many companies or research institutes have built and published large-scale knowledge graphs in recent years. Based on the construction methods and data sources, the knowledge graphs published by different laboratories vary. The earliest knowledge base project, Cyc, is a manually constructed common-sense knowledge graph, which encodes information from the common-sense knowledge base into a form that can be understood and used by machines for intelligent inference through manual methods.

Knowledge graphs can help machines understand a domain and thus improve the understanding of models, and understanding a domain often starts with understanding the domain vocabulary, which is the key knowledge for understanding user intent. For example, a recruiter who is looking for experts or scholars in the domain of knowledge mapping only needs to determine whether the candidate's resume or thesis title contains the domain vocabulary of knowledge mapping. The rule, dictionary, and online knowledge base-based approach is a traditional manual approach where many domain experts perform manual writing of rules that are used to extract specific words, such as place names and group names. Due to the high cost of manually constructing rules and the limited ability of experts to cover too large a range, the completeness and accuracy of the rule-based entity identification method are not guaranteed under the current situation where data are updated and iterated too quickly. Corporate social capital is the collection of explicit and potential resources, and capabilities embedded in the structure of a firm's network that can be controlled by the firm, are based on trust and reciprocity, contribute to the quality and efficiency of organizational processes, and facilitate the achievement of the firm's objectives and goal-achieving activities. It is also said that corporate social capital is the ability of a firm to take in scarce resources through vertical links, horizontal links, and social connections.

The financial situation is closely related to the business situation. Some scholars in academia have studied the relationship between service innovation and corporate financial performance, and they have classified service innovation from different perspectives such as service concept and customer meeting, and based on this, they have quantified and concluded that service innovation will improve the financial performance of the company to a great extent. They found that there is a lag in innovation investment, the initial impact brought by the investment is negative, and only after a long period will it bring good economic benefits to the company, this is because innovation is inherently uncertain, while the market changes in a variety of ways, innovation to better integrate consumer needs requires long-term, high-quality research, and development activities.

(1) $$\begin{array}{c} \left[\left[\text{CLS}\right],w_{1}^{r},w_{2}^{r},w_{3}^{r}\ldots ,w_{n}^{r}\right]=\text{SEP.} \end{array}$$

The cost of retaining resources rises along with the increased uncertainty of economic policies, and the cost of adjusting downward to resources will fall at the same time. This makes the costs much less sticky, so companies want to get better and generally reduce resources and costs when economic policies are in uncertainty, to minimize the risk of economic uncertainty as much as possible. Therefore, from a resource perspective, it also hurts the social capital of enterprises [17]. There is an inverse relationship between economic policy uncertainty and firms' investment; that is, firms are more inclined to reduce their investment behavior and promote the collection of resources when uncertainty is raised, which has an impact on the sustainable maintenance of internal and external social capital relationships of firms.

(2) Or=Re LUhCLS2·WRD−bRD.

In general, heterogeneity refers to the variance that exists across studies. Whether or not each sample falls within the same distribution is the fundamental purpose of the test, and is an important part of the process that must be undergone to integrate a single effect size into a composite effect size. Sources of heterogeneity are abundant and include different designs among studies, different lengths of follow-up, the presence of different moderating variables or different moderation, differences in statistical methods or models among studies, and differences in study quality. The choice of which model to analyze is also determined by the results of heterogeneity.

(3) $$\begin{array}{c} P=\text{soft }\max\left(O^{r}\right). \end{array}$$

When heterogeneity is evident, a random-effects model that considers both within-study sampling errors and incorporates between-group variance is selected. This is because the random-effects model will be corrected for the weights given to the studies included in the meta-analysis, and the weights of studies with small (large) sample sizes will be larger (smaller) than in the fixed-effects model, to balance the problem of uneven weight distribution that arises when there are large differences in sample sizes between studies and make the meta-analysis results more accurate. However, the model typically produces higher variance and wider confidence intervals, making it more difficult to explain the reasons for differences. When the value of heterogeneity is relatively small or the cause is clear, then a fixed-effects model is considered a more appropriate choice.

(4) $$\begin{array}{c} P_{i}^{r}=\frac{\exp\left(O_{i}^{r}\right)}{\sum_{j=1}^{m}\exp\left(O_{j}^{r}\right)}. \end{array}$$

In the prediction stage, the final binary output value *y* can be obtained.

(5) $$\begin{array}{c} y=\arg\min P_{i}^{r}. \end{array}$$

The two methods often used to determine the existence of heterogeneity are the *Q* test and the *I*^2^ test. The *Q* test chosen for this study is also known as the chi-squared test, which calculates the *Q* statistic that follows a chi-squared distribution based on the overall squared error of effect values.

**Figure 2:**
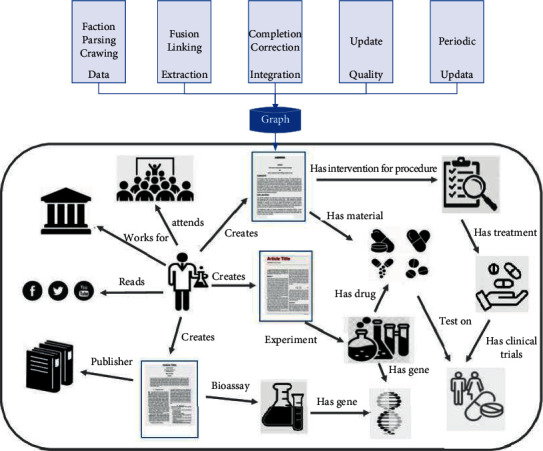
Knowledge mapping association model based on cross-attention units.

The government controls public resources, including operational and innovation resources necessary for the development of enterprises, and it also holds the power to allocate these resources, approving key projects and allocating scarce resources rationally [18]. Currently, China's market mechanism and related institutional system are still imperfect, and the government intervenes in the market to a certain extent to optimize resource allocation, including the use of administrative approval and other forms of control over scarce resources, and the establishment of access rules for some industries. If enterprises want to maintain a leading position in the market competition, improve their core competitiveness, and maximize their market value, they must obtain scarce resources from the government, as shown in Figure 2.

For example, if the number of samples with positive true labels is particularly small, accounting for only 2% of the total number, then, if the predicted results of the model are all negative, the final accuracy can still reach a high level of 98%. However, the model is very bad. Trust is a major social capital that determines a country's economic growth and social progress, in addition to physical and human capital. At the macro level, the level of trust in a country plays an important role in its long-term social stability and sustained economic growth, as well as in the improvement of economic efficiency. At the micro level, a higher level of trust within and between enterprises can increase their competitiveness and improve their performance.

Innovation is a strategic tool to help companies break through bottlenecks and transform themselves in the face of a diverse market environment. Performance is the feedback to the enterprise organization, team, or individual to achieve the goal. From a comprehensive viewpoint, enterprise innovation performance is the feedback to measure the efficiency and excellence of enterprise innovation activities and is a comprehensive indicator to evaluate the degree of excellence of enterprise innovation activities. The definition of enterprise innovation performance as the performance of enterprise's R&D input and innovation results has not been unified yet, and a review of related literature reveals that it is mainly based on two perspectives of innovation output and input [19]. The innovation output perspective considers innovation performance as a production-related, innovative product whose output is novel and useful and can bring revenue to the enterprise. The innovation input perspective is the extent to which a firm's innovative products are put into the market, broadly speaking from the formation of the innovation concept to the achievement of the innovation results and their introduction into the market.

Due to the plethora of combinations, only a few of the best-performing combinations of these strategies are reported here. For a multi-input conditional-factual dual-output model using all four types of input sequences, this semisupervised design improves accuracy from 65.09% to 67.37%, the recall from 60.24% to 64.36%, and *F*1 value from 62.57% to 65.82% for label prediction. In tuple cell extraction, it increases the accuracy from 69.51% to 71.42%, the recall from 70.10% to 72.08%, and the *F*1 value from 69.80% to 71.75%.

## 4. Model Design for Correlation Analysis between Social Capital and Innovation Performance

Innovation is a key factor for companies to gain core competitiveness, and it is a strategic choice with risk and uncertainty. The board of directors, as the main decision-making body of the enterprise, uses its rich experience, skills, knowledge, and social relationships to perform the function of supervising the enterprise, establish a network of relationships in the external environment of the enterprise, and provide suggestions for the formulation of corporate strategies and the direction of business development; based on the resource dependence theory, the members of the board of directors of the enterprise can use their internal and external resources to improve the innovation performance of the enterprise through effective ways such as investing in knowledge-based talents and resources [20]. This requires directors to use social capital to obtain resources to support the chosen innovation strategy and to provide legal basis and advice for the formulation of corporate strategies and help companies to obtain external resources. Innovation performance is defined as the increase in firm value following the implementation of the adoption of a new technology, measured as the increase in the amount of business done by the firm. This study uses organizational size as a control variable. It is expected that organizational technological diversification, technology accumulation, and external learning opportunities are likely to have a positive relationship with organizational size. Performance is a multidimensional construct, and the results will vary depending on the factors measured. The United States was the first country to conduct research on innovation performance indicator systems.

**Figure 3:**
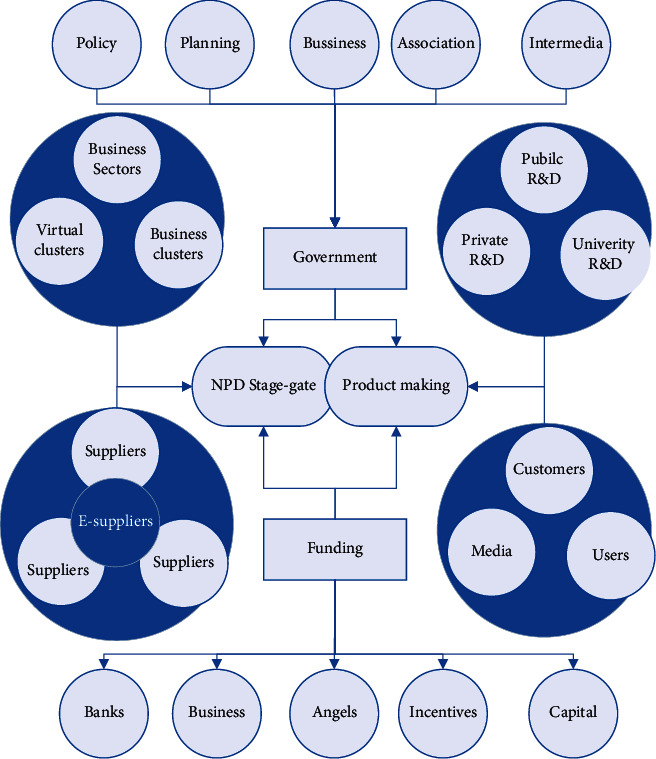
Case study logic and steps.

According to the resource-based view, the resources of different firms are heterogeneous and do not flow freely. For enterprises to obtain differentiated resources, the board of directors is the key factor, social capital has a value that is difficult for other individuals to imitate, board social capital is scarce, and board members of different enterprises have different characteristics that cannot be shared by many enterprises, the formation of board social capital is a long-term accumulation process, and the characteristics of board social capital are also causally ambiguous and have difficult to imitate unique characteristics. This helps companies gain a competitive advantage and industry position. Directors' social capital changes the way directors perform their functions and has a significant impact on corporate innovation strategy decisions as well as the implementation process, as shown in Figure 3.

First, a detailed research and interview outline was developed according to the research objectives of this paper (including questions about the business status, historical events, and partners), which was submitted to the directors of the target companies through an intermediary for review, and after removing research questions involving commercial secrets, the final interview was determined through repeated consultations. Then, we agreed with the person in charge of the company on the interview location and interviewees and determined the interview time without disturbing the normal operation of the company. Finally, semistructured interviews were conducted, each lasting about 2 hours, with senior management, project managers, channel partners, or functional employees. If necessary, additional research was conducted via online video to ensure the completeness of the information. Secondary data were collected from public information of the target companies, and the main sources included the company's official website, WeChat articles, industry information, research books, media reports, research reports, internal company publications, and company publications.

Reputation is an intangible asset that is difficult to be replicated by other companies, which reflects the recognition of the company by society and can help the company achieve sustainable competitive advantage. The good reputation of an enterprise can help it obtain scarce resources, development opportunities, and related support. Corporate reputation provides support for enterprises in forming competitive advantages in the market, maintaining core competitiveness, and enhancing market position, and broadening the development space of enterprises [21]. By maintaining a good reputation, enterprises can gain more investors and highly qualified talents, thus relieving their financial pressure and ensuring the vitality of their development.

The complexity of R&D activities makes it impossible for enterprises to rely solely on their internal resources to carry out technological innovation activities. Enterprises need to obtain innovative scientific and technological information and innovation resources from outside to support their internal R&D activities. The social capital owned by an enterprise reflects the enterprise's reputation. Good social capital enables an enterprise to receive certain government policy support and subsidies and, at the same time, reduces the difficulty of obtaining loans. The larger the stock of social capital of an enterprise, the more external network resources of government and financial institutions the enterprise can access, and thus obtain more credit funds to relieve its financial pressure in the process of carrying out R&D activities. Corporate reputation is also a reflection of the quality of enterprise development. Nowadays, China's market mechanism is still not sound, and credit institutions will not only examine the financial information of enterprises but also their reputation in the process of lending, and credit institutions will evaluate the quality of enterprise development based on these two indicators. Data modeling is a process used to define and analyze the requirements of data and the corresponding information systems it needs to support. There are three different types, from requirements to the actual database. The data model used for information systems as a conceptual data model is essentially a set of initial specification techniques for documenting data requirements. Therefore, in today's market mechanism, which is still improving, an enterprise with a good reputation can gain the favor of credit institutions and reduce the shortage of funds, which allows more funds to be invested in R&D activities.

Enterprises need to have the latest technical and financial information to make the right R&D decisions, but the existence of information asymmetry makes it difficult for enterprises to obtain the latest information, thus restricting their R&D activities. Companies with high social capital stock can use their social capital to ensure communication with external organizations and broaden their information access so that they can learn the latest information and lift the limitation caused by information asymmetry [22]. Therefore, social capital can help enterprises reduce the negative impact of information asymmetry, reduce financing costs, and support their R&D investment decisions.

According to the theory of technological innovation, innovation is an investment activity with high risk, and R&D activities often require a large investment of capital and workforce and have a high chance of failure. From the beginning of innovation investment to the final mass production of innovative products or the application of innovative technologies, there are uncertainties and uncontrollable risks in each step of innovation activities. Enterprises can use the social capital they have to cooperate with external organizations for innovation, and avoid the risks in innovation activities by sharing innovation resources, sharing innovative information, and building innovation platforms. A knowledge graph is essentially a knowledge base called a semantic network, that is, a knowledge base with a directed graph structure, where the nodes of the graph represent entities or concepts and the edges of the graph represent the various semantic relationships between entities/concepts.

## 5. Analysis of Results

### 5.1. Performance Analysis of Knowledge Graph

**Figure 4:**
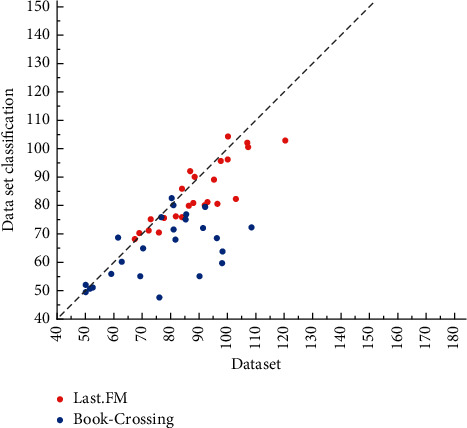
Hyperparameter.

The experiments in this paper are tested in the same hardware environment and the same framework, and the code is modified in the operating system using the editor, and the model is tuned and optimized. In this experiment, the hyperparameters of the benchmark model are the same as its original settings, and the hyperparameters set by CAKR concerning the three datasets are shown in Figure 4. For different datasets, different embedding dimensions, regularization coefficients, and learning rates of the two tasks are used, and the datasets used are divided into training, validation, and test sets in the ratio of 6 : 2:2, and the results are observed in several experiments. Finally, the average of the prediction results of the valid experiments is taken to verify the robustness.

CKE first extracts the semantic representation of each item from the knowledge graph, text description, and visual image to jointly learn the implicit vector of collaborative filtering. Here, since text descriptions and visual images are not available in the dataset, each item is only represented as an embedding learned by transfer, but the transfer is more suitable for applications within mapping tasks rather than recommendation tasks. This suggests that the model can learn user biases accurately when there are enough records of user behavior and sufficient interaction data available. However, the quality and size of the dataset affect RippleNet more, and the experimental results show that the model does not perform as well as MKR on the book and music datasets.

For the multitask learning framework, the original model MKR has the best overall performance compared with other benchmarks, which proves the effectiveness of the multitask learning framework and alternate learning; Ripp-MKR model is a fusion of RippleNet and MKR model, and by introducing the concept of ripple propagation, MKR model can dig deeper into the connection between items and improve the logic of the knowledge graph structure. The model structure is more complex and increases the computational cost.

**Figure 5:**
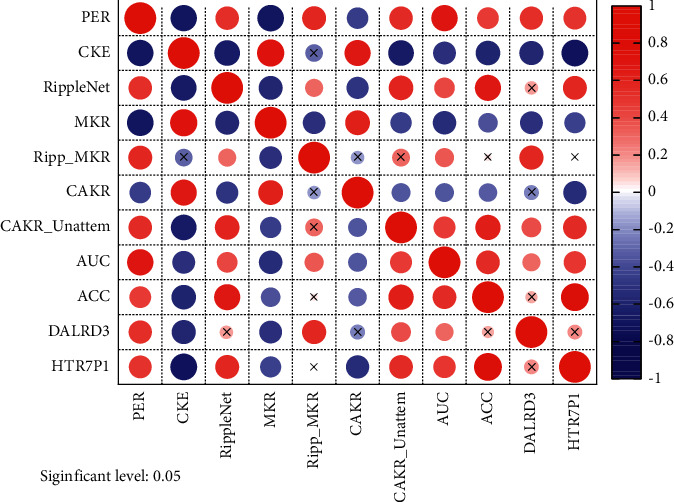
Experimental comparison results.

The model CAKR optimizes the core part of the multitask learning framework, which is not achieved by Ripp-MKR. The AUC score improves by 0.8% on the movie dataset and surpasses RippleNet, which is not achieved by MKR; it improves by 1.2% on the book dataset and 0.6% on the music dataset. The ACC score metric also has some improvement on the three datasets, as shown in Figure 5.

If the multi-output model has both RNT and TCT layers, the F1 value is 1.4–5.0% higher than that of the model with only the RNT layer. Moreover, the recall is relatively higher by 1.5–9.0%. Thus, the TCT layer that generates multiple sequences of labels for each tuple type (i.e., facts and conditions) plays a very important role in identifying multiple tuples from statements.

**Figure 6:**
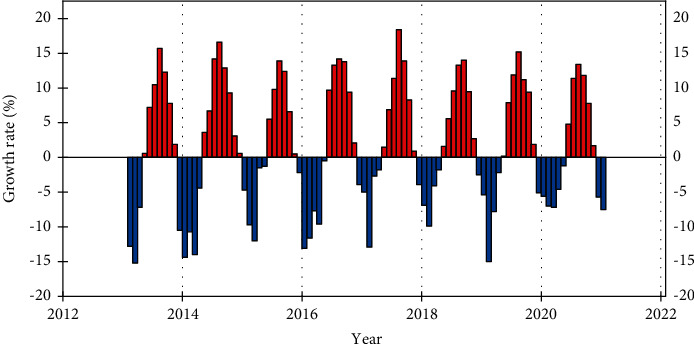
Experimental results of objective evaluation.

This is an implicit conditional knowledge graph representation learning algorithm that is extremely generalizable and efficient, and the implicit vectors resulting from representation learning can be used for numerous downstream application tasks and are not limited to the literature search in this chapter. In this work, a BLS system based on conditional knowledge graph representation learning is proposed, with a core architecture of multi-encoder for incorporating multilayer conditional knowledge graphs (multiknowledge) obtained from text structuring, called MEMK. MEMK has two core modules corresponding to two uses of the multiknowledge graph: a query extension module and a representation learning module. For the query extension module, MEMK matches the internal knowledge of a query with the set of documents to obtain factually relevant concepts to extend the original query. In the representation learning module, MEMK models for the four types of knowledge information are using multiple stacked transformer encoder layers, as shown in Figure 6. Market allocation of resources refers to the process of allocating, combining, redistributing, and recombining resources in accordance with changes in market demand and supply caused by price changes in the course of economic operation. Market allocation of resources is mainly carried out through price, supply and demand, competition, etc.

The research is oriented to the application techniques of conditional knowledge mapping with strong interpretation and generalization. The key points of the study are how to explicitly portray knowledge matching in conditional knowledge graphs and how to learn the proposed hierarchical conditional knowledge graphs layer by layer and facilitate the use of the graphs for other downstream tasks. To address these two key points, we propose a document search algorithm based on conditional knowledge map critical path matching, which is highly interpretable and can match directly at the knowledge level and will give the required conditional information for matching to the target factual knowledge. The literature search algorithm is based on conditional knowledge graph representation learning, which based on learning model with strong generalization, and the resulting implicit vector can be used for any downstream application tasks and is not limited to the literature search.

### 5.2. Analysis of Correlation Analysis Model Test Results

**Figure 7:**
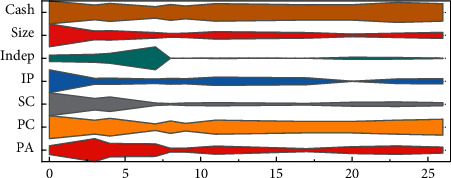
Descriptive statistical characteristics of key variables.

The study conducted a descriptive statistical analysis of the valid sample data, and the results are shown in Figure 7. From the table of descriptive statistical analysis, the mean value of innovation performance of the explanatory variable enterprises is 2.06 and the standard deviation is 1.31.

Since different industry types have different needs for innovation results, based on the above data, there are significant differences in innovation performance among different enterprises. For the explanatory variables, there are negative values due to standardization during data processing. The mean value of Board Social Capital is 1.170; the mean value of Board Business Ties is 0.85; the mean value of Board Political Ties is −0.24; and the mean value of Board Academic Background is 0.26. It can also be seen from the table that the minimum value of Board Social Capital is −3.170 and the maximum value is 10.01, with a standard deviation of 2.590, which indicates that the social capital of the board of directors varies significantly among enterprises. The standard deviation of the board of directors' business ties is 1.550, which indicates that there is a certain degree of difference in board of directors' business ties; the standard deviation of the board of directors' political ties is 1.440, which indicates that there is a difference in political ties of the board of directors among different enterprises; the standard deviation of the board of directors' academic background is 0.260, with a maximum value of 0.710 and a minimum value of 0. The value of the board of directors' academic background is taken to be between 0 and 1.

Among the control variables, the mean value of the proportion of independent directors is 0.730, the maximum value is 2.250, the minimum value is 0.25, and the standard deviation is 0.5; the mean value of the return on assets is 0.040, the maximum value is 0.190, the minimum value is -0.110, and the standard deviation is 0.050, which shows that the enterprises are relatively balanced. The standard deviation of the gearing ratio is 0.20, the minimum value is 0.050, and the maximum value is 0.860, with a large extreme difference, which is influenced by the type of enterprises and leads to a large difference in the level of indebtedness among different enterprises; the mean value of enterprise cash flow is 0.040, and the standard deviation is 0.060, which indicates that the cash flow is less volatile, and the mean value of enterprise nature is 0.680, which is greater than 0.5 and indicates that the non-state-owned enterprises are less volatile. The mean value of enterprise nature is 0.680, which is greater than 0.5, indicating that there are more nonstate enterprises.

**Figure 8:**
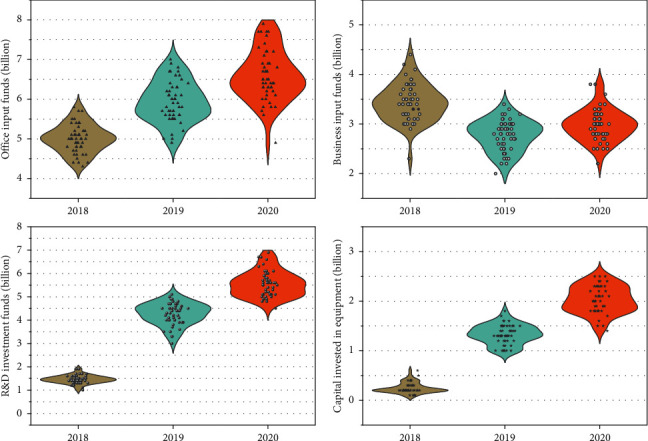
R&D capital investment and lagged one-period technological innovation performance.

In this paper, to verify the correlation between R&D investment and technological innovation performance, the line graph is used to show the two, and the relationship between them is judged according to their trends. The line graphs of total R&D personnel input, input intensity, and lagged period technological innovation performance from 2015 to 2018 are shown in Figure 8, the total R&D personnel input and input intensity of JFST from 2015 to 2018 generally show an increasing trend and the lagged period of the same period. In 2017, because the total number of employees increased from 6717 to 7309, which is a large increase, but the R&D personnel did not show a significant increase, so there is a decreasing trend of R&D personnel input intensity in 2017, and because R&D is a long process and the cycle of the R&D process is long, the R&D personnel's R&D results often have a certain lag, and the R&D investment made in the early stage often has to be delayed for several periods before it can generate benefits. Only after an enterprise's technology stock has accumulated to a certain scale, the market recognition of its products and its market share will be qualitatively improved, and the initial R&D investment will only really create benefits at this time. Therefore, the upward trend of JFST's technological innovation performance is not affected by the slight decrease in its R&D personnel investment intensity in 2017. Combined with the data analysis, the relationship between R&D personnel investment and technological innovation performance of JFST shows a positive correlation.

Total R&D capital investment, investment intensity, and technological innovation performance show a positive correlation. As JFST keeps increasing the total amount of R&D capital investment, its technological innovation performance also keeps improving. This indicates that technology has obvious output performance for the capital invested in R&D activities, and the company has high R&D efficiency and can successfully convert R&D results into economic benefits.

## 6. Conclusion

The complexity of R&D activities makes it impossible for enterprises to rely solely on their internal resources to carry out technological innovation activities. Enterprises need to obtain innovative scientific and technological information and innovation resources from outside to support their internal R&D activities. The social capital owned by enterprises reflects their reputation. In today's market mechanism is still being improved, enterprises with a good reputation can gain favor from government and credit institutions, obtain policy and financial support, reduce the occurrence of capital shortage, and enable more capital to be invested in R&D activities. Social capital plays a part in mediating the relationship between economic policy uncertainty and firms' innovation performance. From the empirical results, the mediating effect of social capital is about 0.8%. The reason is that the accumulation of social capital is a necessary capital expenditure item for enterprises to maintain daily operation, and social capital involves more internal and external social capital, covers a wide range of areas, occupies more capital of enterprises, and takes a longer period to generate benefits. Therefore, to avoid the negative impact of this situation, enterprises tend to reduce the maintenance cost of social capital and change the purpose of investment; that is, they prefer to invest in R&D and innovation activities of enterprises to improve the efficiency of capital use, improve technological products, and guarantee the economic expectation of enterprises. To avoid the negative impact of this situation, firms tend to reduce the cost of maintaining social capital and change the purpose of their investments; that is, they prefer to invest in R&D and innovation activities to improve the efficiency of capital use, increase the level of technological product innovation, and ensure the competitiveness of firms through R&D and innovation even in times of poor economic expectations.

## Data Availability

The data used to support the findings of this study are included within the article.

## References

[1] Miković R., Petrović D., Mihić M., Obradović V., Todorović M. (2020). The integration of social capital and knowledge management - the key challenge for international development and cooperation projects of nonprofit organizations. *International Journal of Project Management*.

[2] Á M. P., Elche D., García-Villaverde P. M., Parra-Requena G. (2019). Cultural tourism clusters: social capital, relations with institutions, and radical innovation. *Journal of Travel Research*.

[3] Östh J., Dolciotti M., Reggiani A., Nijkamp P. (2018). Social capital, resilience and accessibility in urban systems: a study on Sweden. *Networks and Spatial Economics*.

[4] Raffiee J., Byun H. (2020). Revisiting the portability of performance paradox: employee mobility and the utilization of human and social capital resources. *Academy of Management Journal*.

[5] Choi H.-J., Ahn J.-C., Jung S.-H., Kim J.-H. (2020). Communities of practice and knowledge management systems: effects on knowledge management activities and innovation performance. *Knowledge Management Research and Practice*.

[6] Peiró-Palomino J. (2019). The geography of social capital and innovation in the European Union. *Papers in Regional Science*.

[7] García-Villaverde P. M., Parra-Requena G., Molina-Morales F. X. (2018). Structural social capital and knowledge acquisition: implications of cluster membership. *Entrepreneurship & Regional Development*.

[8] Wang Z., McNally R., Lenihan H. (2019). The role of social capital and culture on social decision-making constraints: a multilevel investigation. *European Management Journal*.

[9] Eriksson R., Rataj M. (2019). The geography of starts-ups in Sweden. The role of human capital, social capital and agglomeration. *Entrepreneurship & Regional Development*.

[10] Neneh N. B. (2018). Family-work conflict and performance of women-owned enterprises: the role of social capital in developing countries--implications for South Africa and beyond[J]. *Journal of International Women’s Studies*.

[11] Lane A. P., Wong C. H., Močnik Š, Song S. B. (2020). Association of neighborhood social capital with quality of life among older people in Singapore. *Journal of Aging and Health*.

[12] Benbow R. J., Lee C. (2019). Teaching-focused social networks among college faculty: exploring conditions for the development of social capital. *Higher Education*.

[13] Nawinna D., Venable J. R. (2019). Effects of ICT-enabled social capital on inter-organizational relationships and performance: empirical evidence from an emerging economy. *Information Technology for Development*.

[14] Sadri A. M., Ukkusuri S. V., Lee S., Clawson R. D. M. S. J. D. (2018). The role of social capital, personal networks, and emergency responders in post-disaster recovery and resilience: a study of rural communities in Indiana. *Natural Hazards*.

[15] Suzuki K., Demircioglu M. A. (2019). The association between administrative characteristics and national level innovative activity: findings from a cross-national study. *Public Performance and Management Review*.

[16] Hu A., Wu X. (2019). Science or liberal arts? Cultural capital and college major choice in China. *British Journal of Sociology*.

[17] Hamilton M. L., Lubell M. (2019). Climate change adaptation, social capital, and the performance of polycentric governance institutions. *Climatic Change*.

[18] Rodríguez-Pose A., Lee N., Lipp C. (2021). Golfing with Trump. Social capital, decline, inequality, and the rise of populism in the US. *Cambridge Journal of Regions, Economy and Society*.

[19] Muafi M. (2020). A nexus among strategic orientation, social network, knowledge sharing, organizational innovation, and MSMEs performance. *The Journal of Asian Finance, Economics and Business*.

[20] Prasetyo P. E., Dzaki F. Z. (2020). Institutional performance and new product development value chain for entrepreneurial competitive advantage. *Uncertain Supply Chain Management*.

[21] Celata F., Sanna V. S. (2019). A multi-dimensional assessment of the environmental and socioeconomic performance of community-based sustainability initiatives in Europe. *Regional Environmental Change*.

[22] Koranteng F. N., Wiafe I., Kuada E. (2019). An empirical study of the relationship between social networking sites and students’ engagement in higher education. *Journal of Educational Computing Research*.

